# Fractal Electrodes as a Generic Interface for Stimulating Neurons

**DOI:** 10.1038/s41598-017-06762-3

**Published:** 2017-07-27

**Authors:** W. J. Watterson, R. D. Montgomery, R. P. Taylor

**Affiliations:** Physics Department, University of Oregon, Eugene, OR 97403 USA

## Abstract

The prospect of replacing damaged body parts with artificial implants is being transformed from science fiction to science fact through the increasing application of electronics to interface with human neurons in the limbs, the brain, and the retina. We propose bio-inspired electronics which adopt the fractal geometry of the neurons they interface with. Our focus is on retinal implants, although performance improvements will be generic to many neuronal types. The key component is a multifunctional electrode; light passes through this electrode into a photodiode which charges the electrode. Its electric field then stimulates the neurons. A fractal electrode might increase both light transmission and neuron proximity compared to conventional Euclidean electrodes. These advantages are negated if the fractal’s field is less effective at stimulating neurons. We present simulations demonstrating how an interplay of fractal properties generates enhanced stimulation; the electrode voltage necessary to stimulate all neighboring neurons is over 50% less for fractal than Euclidean electrodes. This smaller voltage can be achieved by a single diode compared to three diodes required for the Euclidean electrode’s higher voltage. This will allow patients, for the first time, to see with the visual acuity necessary for navigating rooms and streets.

## Introduction

**Figure 1:**
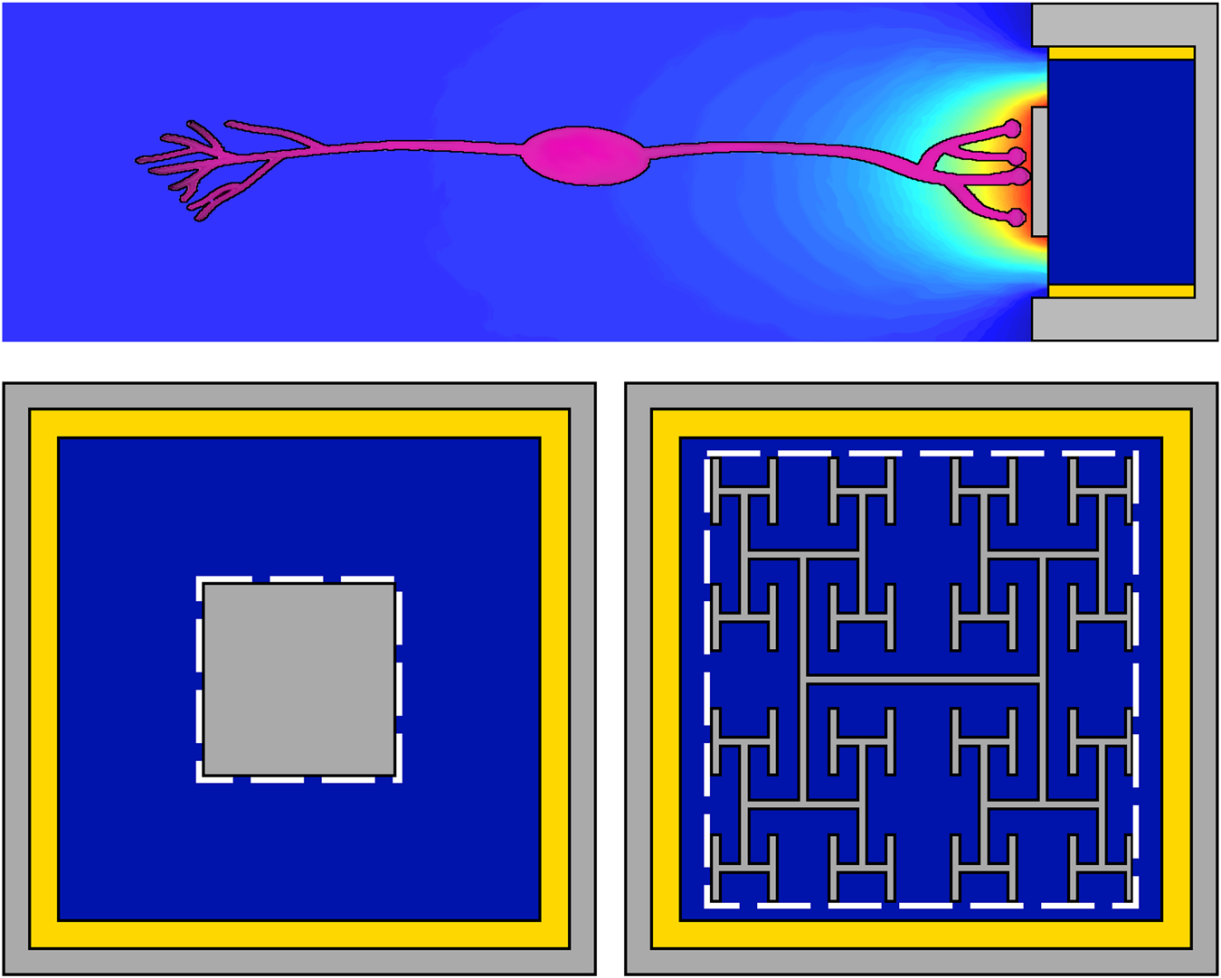
The subretinal implant design. (Top) Side-view. Light passes through the retinal layer of neurons (pink) to reach the photodiode (dark blue). The photodiode then generates a voltage difference between its two electrodes (grey) which are separated by an insulating region (yellow). (Bottom-left) Top view of the traditional design featuring a square inner electrode. (Bottom-right) Top view of our fractal inner electrode featuring a repeating H design. In both cases, the dashed white line indicates the bounding perimeter of the inner electrode.

The emotional and economic impact of vision loss is staggering. According to the Brightfocus Foundation, the annual global cost of retinal diseases is in excess of $340 billion^1^. This has triggered the development of retinal implants to restore vision to victims of retinal diseases such as macular degeneration and retinitis pigmentosa^2–9^. Human clinical trials have restored visual acuity up to 20/1260 for epiretinal implants^2^ (positioned in front of the retina) and up to 20/546 for subretinal implants^3, 4^ (positioned at the back). However, the latter was observed in only one patient; for 86% of patients, the visual acuity wasn’t restored to a measurable level. Subretinal implants used in the clinical trials featured an array of 1500–5000 artificial photoreceptors fabricated on a 2–3 mm silicon chip, which was inserted into the retinal region where photoreceptors had been damaged^3–5^. A conventional implant architecture is summarized in Fig. 1. A p-n photodiode receives light and generates an electrical field between the inner and grounded outer electrodes. If located close enough to experience this field, the retina’s bipolar neurons are stimulated and pass their signal via ganglion neurons down the optic nerve to the brain’s primary visual area^10, 11^.

Here, we propose an inner electrode that exploits fractal geometry rather than the Euclidean shapes used in today’s implants (Fig. 1). Fractals are prevalent in nature, in part because of their ability to generate a large surface area within a given volume^12^. For example, this allows bronchial trees to transfer oxygen to the bloodstream, trees to absorb sunlight, and coastlines to disperse wave energy. Fractal electrodes’ large surface area increases their capacity to hold electrical charge, which in turn generates large electric fields. Our fractal electrodes feature ‘branching’ patterns that repeat at different size scales, similar to the neuron dendrites they interface with^13, 14^. These fractals contrast with other electrodes which feature a fractal ‘mountain’ roughness^15, 16^. In addition to their enhanced fields, both types of fractal electrode are expected to promote neural adhesion. Experiments demonstrate that neurons adhere preferentially to textured surfaces^17–19^. In particular, they extend their neurites along the texture established by edge patterns^20–22^. Because our fractal branch design maximizes the density of electrode edges compared to Euclidean geometries, the resulting texture might increase neural adhesion. Their resulting proximity to the electric field would then ensure high stimulation rates.

Our fractal branch design offers two more advantages for retinal implants that are absent for fractal mountain electrodes. Firstly, the gaps between the fractal branches allow light to pass into the underlying photodiode. Studies unrelated to implants have shown that the gaps in fractal electrodes exhibit extraordinary transmission of electromagnetic radiation^23, 24^ (i.e. the transmitted radiation is greater than a simple pixel count of the electrode’s covering area would predict), and that the transmitted wavelength (and therefore color) can be tuned^25, 26^. Adopting a fractal branch design for implants could therefore result in enhanced light sensitivity. Secondly, fractal structures are mechanically conformal^27^, a highly desirable quality for electronics required to match the retina’s curved surface. Based on this potential to integrate their favorable electrical, adhesive, optical, and mechanical properties, here we quantify the superior neural stimulation generated by the enhanced capacitance of branched fractal electrodes compared to Euclidean designs.

## Results

### Electrode Properties

We modelled retinal implants operating with a sinusoidal electrical potential of frequency *f*, $V={V}_{0}{e}^{i2\pi ft}$, applied to capacitive TiN electrodes in retinal fluid. This oscillating potential is necessary to overcome ionic screening by the fluid and can be achieved by modulating the light entering the photodiode^28^. We chose a sine wave oscillation because of its broad applicability. Any waveform, including the square waves typically used in today’s implants^3, 28^, can be constructed from a Fourier sum of sine waves. Demonstration of the superior operation of the fractal electrode for a sine wave will automatically translate to a sum of sine waves and therefore to any waveform. We also note that, for simplicity, we excluded the rest period between pulses which retinal implants employ to minimize visual percept fading^3, 29^. Its inclusion post simulation would not impact any demonstration of superior operation.

The electrode height was 250 nm and the outer dimension of the ground electrode was 20 µm × 20 µm. The three chosen geometries for the inner electrode (fractal, square and grid) had identical covering areas of 50 µm^2^, where covering area is the top surface area of the inner electrode (i.e. the area which blocks incoming light ignoring diffraction and extraordinary transmission). This was done to standardize light transmission. To model the electrodes’ electric fields, three dimensional geometries were meshed and node-to-node impedances were defined using an equivalent circuit model (Methods).

**Figure 2:**
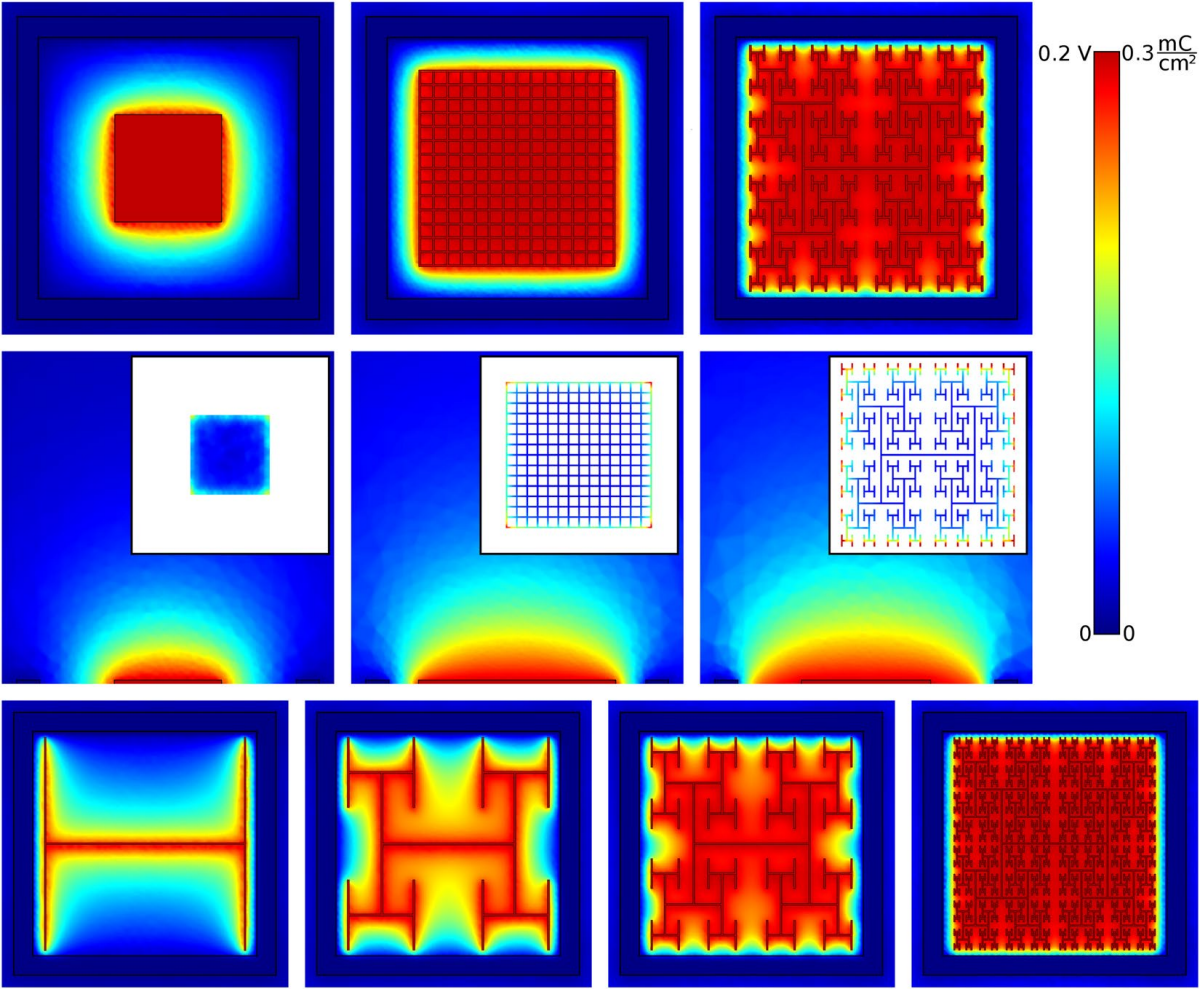
The maximum extracellular voltages reached during each oscillation for the square, grid, and the 3-iteration fractal electrodes (each with an electrode height of 250 nm). In each case, the applied voltage was *V*_0_ = 0.2 V and *f* = 1 kHz. A horizontal slice (at the inner electrode’s top surface) of the three-dimensional voltage distribution for each electrode geometry is shown in the top row. Vertical slices through the middle of the electrodes are shown in the middle row. The charge density on the top surface of each inner electrode is shown in the insets. The bottom row shows the field uniformity achieved by increasing from 0 to 4 iterations.

When a voltage *V* is applied, the electric charge distributes throughout the electrode to minimize the Coulombic energy with the amount of charge set by the capacitance. The geometric contribution to capacitance can be approximated by *C* ~ *A*_*eff*_/*d*_*eff*_, where *A*_*eff*_ is the effective area (i.e. the area available for charge accumulation) and *d*_*eff*_ is the in-plane separation distance between inner and outer electrodes. When the covering area is held constant across the 3 electrode geometries, the multi-sized gaps in the fractal design give rise to a larger bounding perimeter than the grid or the square. This increases *A*_*eff*_ while also reducing *d*_*eff*_, with a net effect of maximizing the capacitance for all frequencies typically used in neural stimulation, 100 Hz–10 kHz. The increase in *A*_*eff*_ results from an inherent interplay of fractal features, as follows. The charge distribution simulations (Fig. 2) demonstrate that much of the charge resides on the bounding perimeter, providing a physical explanation for why the fractal electrode with its large bounding perimeter holds so much charge. Surprisingly, the presence of gaps in the fractal electrode doesn’t reduce *A*_*eff*_ below the Euclidean values. Instead, the gaps generate a large pattern perimeter and the associated vertical side walls supply extra area for charge accumulation. The increased capacity to hold charge leads to the fractal electrode generating an extracellular field which extends further from the electrode surface (Fig. 2). This field penetration is enhanced by the lower resistance of the liquid surrounding the fractal electrode (Supplementary Fig. 1). As expected, the vertical component of the current also penetrates further (Supplementary Fig. 2). With enough fractal iterations, both the field and current density become as uniform as those of the square despite the presence of the light-transmitting gaps (Fig. 2). The field penetration of the grid is intermediary to that of the square and fractal. As with the fractal, the grid also increases its surface area due to the internal side walls. However, the fractal more efficiently utilizes its available area within the 20 μm confined space due to its larger bounding area.

**Figure 3:**
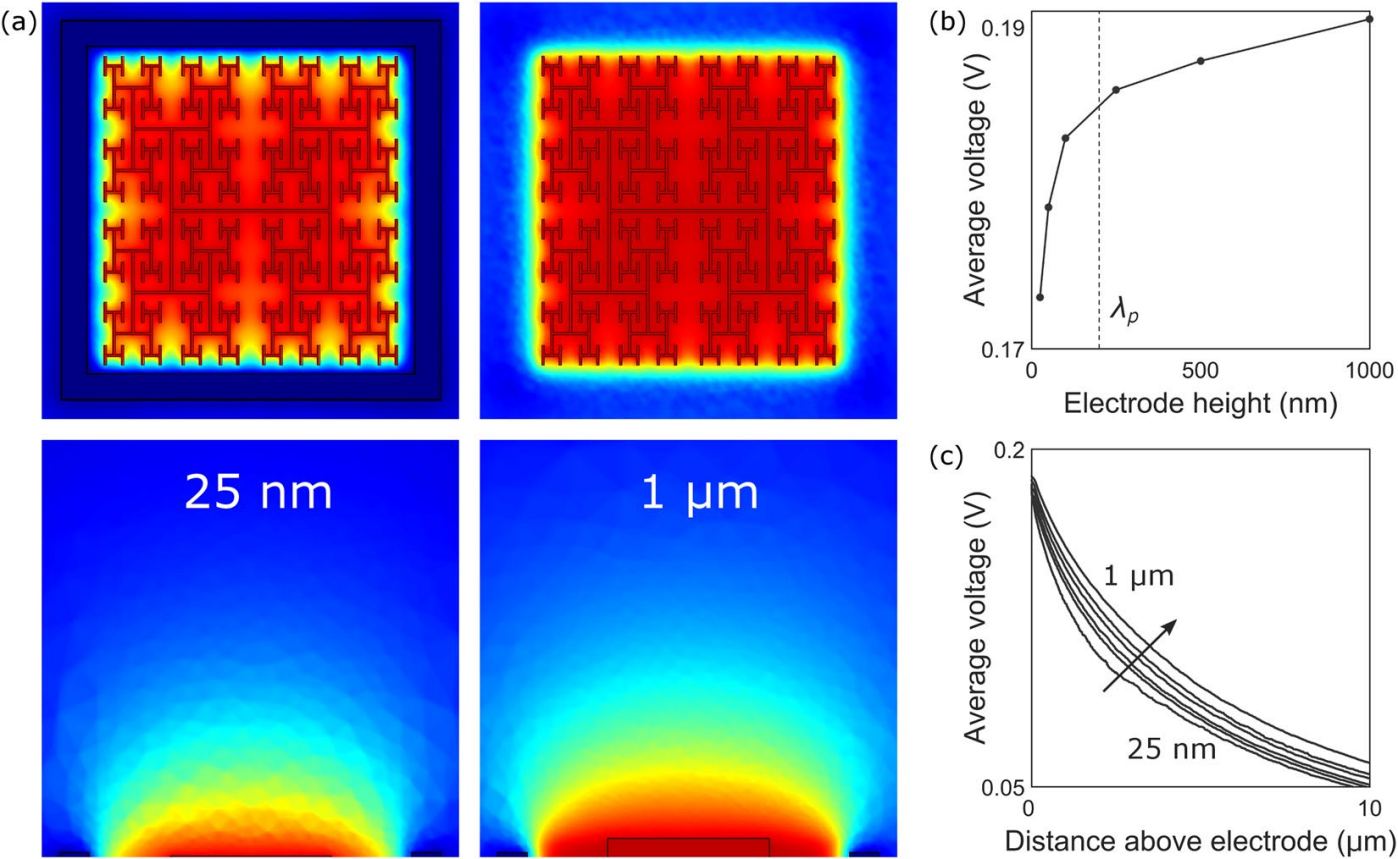
(**a**) Maximum extracellular voltages reached during each oscillation for inner electrode heights of 25 nm (left) and 1 µm (right). In each case, the applied voltage was *V*_0_ = 0.2 V and *f* = 1 kHz. The scale ranges from 0 V (blue) to 0.2 V (red). A horizontal slice (at the inner electrode’s top surface) of the three-dimensional voltage distribution is shown in the top row. Vertical slices through the middle of the electrodes are shown in the bottom row. (**b**) The average voltage at the inner electrode surface (averaged across locations within the bounding perimeter) for varying electrode heights. The penetration depth, *λ*_*p*_, occurs at 200 nm. (**c**) The average voltages plotted as a function of distance above the inner electrode surface for inner electrode heights of 25 nm, 50 nm, 100 nm, 250 nm, 500 nm, and 1 µm (bottom to top).

To optimize the enhanced field penetration achieved by the fractal geometry, we simulated different electrode heights varying from 25 nm to 1 µm. The ground electrode’s height was kept constant at 250 nm in order to isolate the fractal effect. As the fractal’s height is increased, and more charge accumulates on the vertical walls, the field becomes more uniform (Fig. 3). In particular, the average field at the electrode’s surface begins leveling off as the electrode height approaches 250 nm (Fig. 3b). This observed saturation height is confirmed by de Levie’s model^30, 31^, which states that charge accumulation on the gap’s vertical sidewalls switches from using all available area at low gap depths to accumulating predominantly at the top of the sidewalls at large depths. This crossover behavior occurs at the field’s penetration depth into the gap, ${\lambda }_{p}=\frac{1}{4}\sqrt{\sigma d/\pi f{C}_{dl}}$, where *σ* is the electrolytic conductivity, *C*_*dl*_ is the interfacial (double layer) capacitance of the electrode, and *d* is the gap diameter. For the 3 iteration fractal, the largest circle that can be inscribed in the fractal’s gaps has *d* = 1.7 µm, resulting in *λ*_*p*_ = 200 nm. The slight increase in average field as the electrode height exceeds *λ*_*p*_ is due to the continuing charge buildup on the sidewalls at the bounding perimeter. Combined, these two effects lead to the observed increased field penetration as a function of electrode height (Fig. 3c). Although the fractal geometry will therefore increasingly out-perform the square for larger electrode heights, for the remainder of the paper we focus on 250 nm to facilitate a direct comparison with today’s implants.

### Neural Stimulation

**Figure 4:**
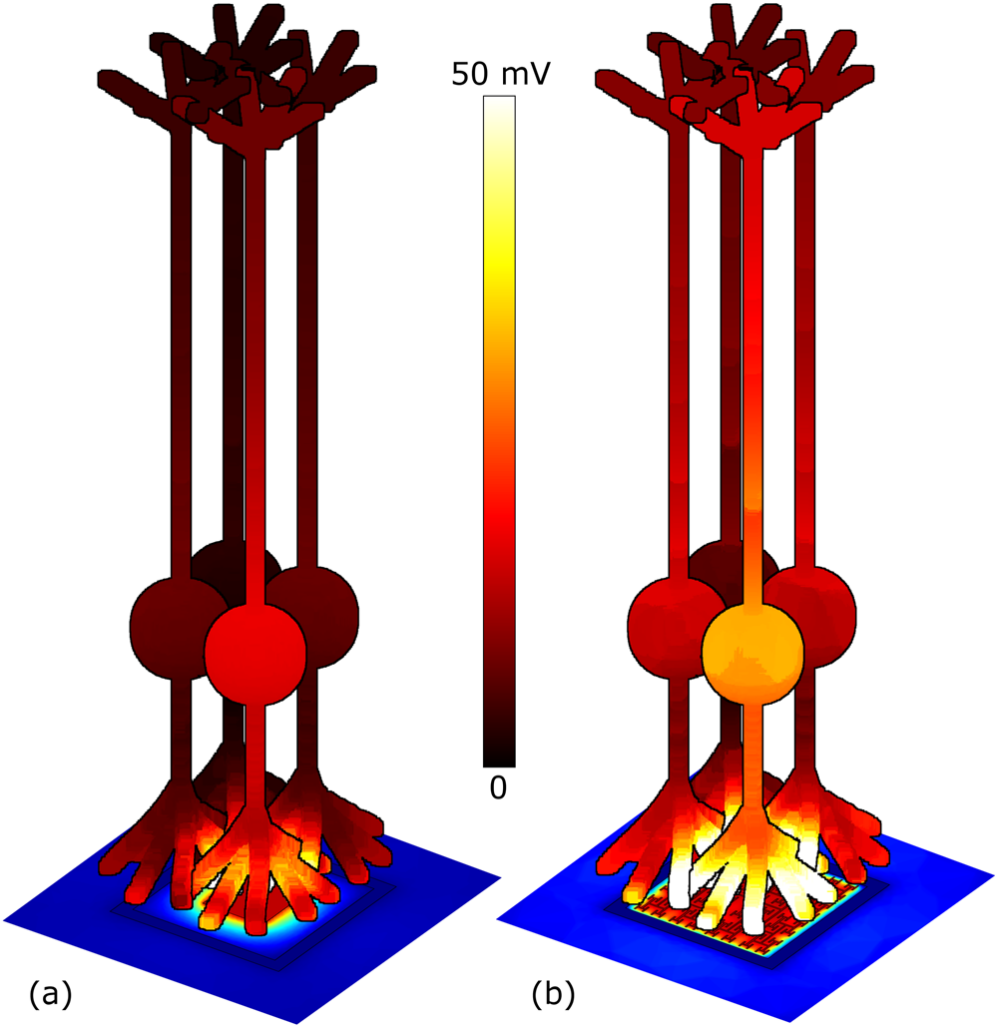
Plot of the maximum Δ*V*_*m*_ (the change of potential across the membrane before and after stimulation) for a patch of bipolar neurons above (**a**) the square and (**b**) fractal electrodes both with *V*_0_ = 0.2 V and *f* = 1 kHz. Maximum Δ*V*_*m*_ within an oscillation is plotted to quantify the greatest stimulation achieved during a cycle. Plots of Δ*V*_*m*_ throughout the electrode’s cycle are shown in Supplementary Fig. 3. For visual clarity, only 4 of the 9 neurons (center and 3 surrounding) are shown. Images are drawn to scale; the neurons are 100 µm in length and the soma is centered 30 µm above the surface.

Because of the enhanced voltage penetration, the fractal electrode induces larger voltage differences across the neuron membranes compared to the square at the same *V* (Fig. 4 and Supplementary Fig. 3). To show this, a patch of 9 bipolar neurons was placed directly above each inner electrode. Voltages obtained from the first part of the simulation were mapped onto the outer membrane of each neuron and an equivalent circuit model was used to solve for the neurons’ internal potentials (Methods). Bipolar neurons have an analog response with depolarization Δ*V*_*m*_ (the change of potential across the membrane before and after stimulation) growing gradually with applied voltage. Previous experiments suggest that the downstream ganglion neurons are stimulated when Δ*V*_*m*_ = 15 mV at the bipolar neuron’s soma^32^. Measuring Δ*V*_*m*_ at the somas, the center neuron (the front most neuron in Fig. 4) above the fractal electrode was depolarized by ~80% more than the center neuron above the square for *V*_0_ = 0.2 V. This larger depolarization for fractal versus Euclidean geometries requires 2 or more iterations (Supplementary Fig. 4).

**Figure 5:**
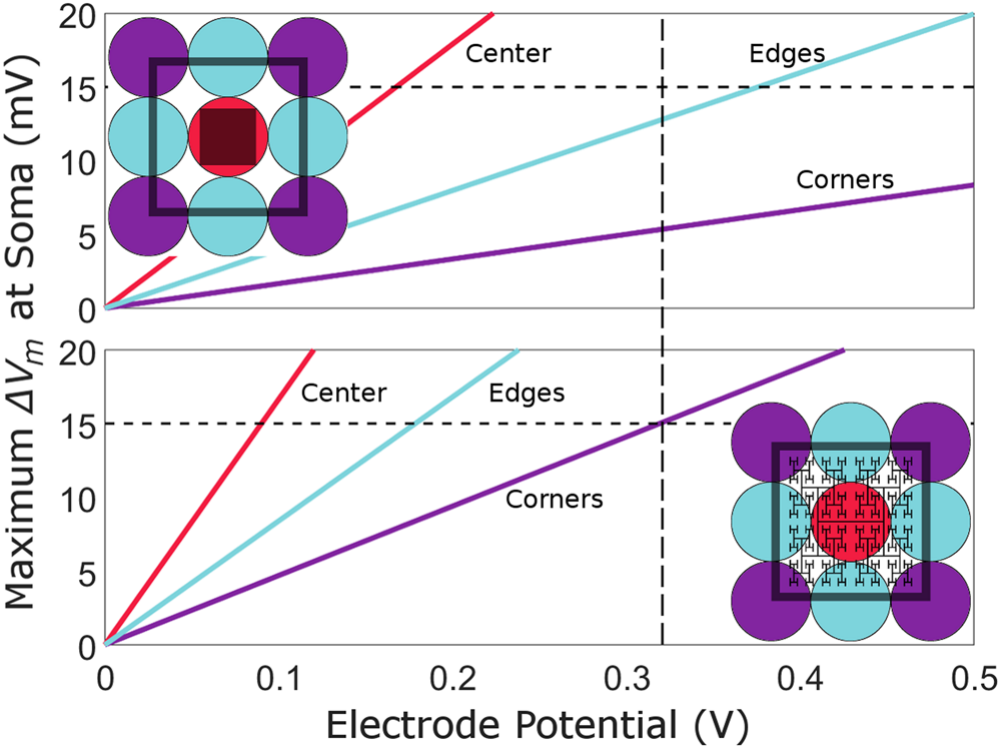
Maximum Δ*V*_*m*_ at the soma plotted against electrode voltage for the 9 bipolar neurons (1 center neuron, red; 4 edge neurons, blue; and 4 corner neurons, purple) above square (top) and fractal (bottom) inner electrodes (each located within an outer electrode of width 20 μm). The vertical dashed line indicates the potential at which all 9 bipolar neurons above the fractal electrode have reached the 15 mV depolarization condition for ganglion stimulation.

To compare stimulation efficiencies, we considered the condition when all 9 bipolar neurons above each electrode (1 center, 4 edges, 4 corners) depolarize by the 15 mV necessary for ganglion stimulation. All 9 neurons above the fractal electrode depolarized by 15 mV for *V*_0_ = 0.32 V while, at this same applied voltage, only the center bipolar neuron above the square met this requirement (Fig. 5). In fact, all 9 neurons above the square did not reach 15 mV depolarization until *V*_0_ = 0.90 V. The voltage required for the grid geometry to stimulate all nine neurons was 0.41 V, intermediary to the fractal and square. We emphasize the general applicability of the above results. The fractal’s superior operation quantified for one sine wave voltage oscillation will be amplified for a summation of sine waves and therefore for the square waves typically used in today’s implants.

## Discussion

We have shown that the branching fractal geometry is an effective approach to increasing the electrode’s capacitance within the confined area of a 20 µm pixel while still facilitating light transmission into the underlying photodiode. Compared to conventional Euclidean geometries, this increased capacitance results in the field penetrating further into the extracellular space and, consequently, an improved stimulation of bipolar neurons. The voltage required to reach the 15 mV depolarization for all 9 neurons above the electrode was 0.90 V for the square but only 0.32 V for the fractal.

This enhanced stimulation holds a number of consequences for subretinal implant operation. Firstly, the fractal voltage resulted in a maximum charge density of only 0.83 mC/cm^2^ on the electrode’s surface. In contrast, at 0.90 V, the charge density at the square’s corners reached 1.01 mC/cm^2^, above the 1 mC/cm^2^ safe charge limit at which TiN electrodes induce hydrolysis^33^. Secondly, the enhanced stimulation influences the visual acuity achieved by the implant as follows. For a typical silicon photodiode (0.6 V open circuit voltage), the fractal voltage can be generated with a single diode of pixel width 20 µm. In contrast, to reach 0.90 V, the square design would require linking 2 or more diodes in series, with current Euclidean designs using 3 photodiodes occupying a 70 µm pixel width^28^. In a simplistic picture, pixel size directly impacts acuity. In natural vision, 20/20 acuity is achieved by resolving 1 arcminute of visual scene, corresponding to a 5 µm pixel at the retina^6^. Assuming acuity scales inversely with pixel size in electronically restored vision, the 70 µm Euclidean design would generate 20/280 and 20 µm fractal would generate 20/80 acuity.

**Figure 6:**
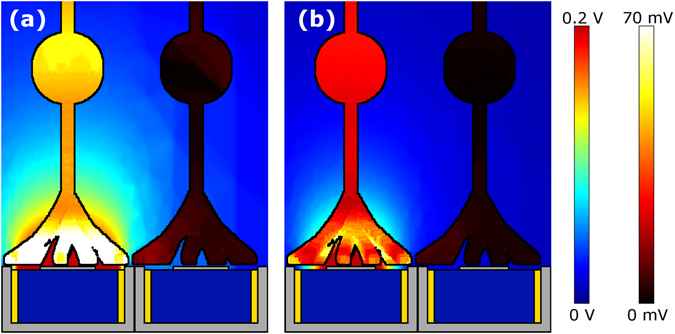
Side-view of neurons above two neighboring photodiodes with (**a**) fractal and (**b**) square electrode geometries. For both geometries, each photodiode features the inner and outer grounded electrodes shown in Fig. 1. In each case, the inner electrode of the left photodiode was biased at *V*_0_ = 0.32 V and *f* = 1 kHz, while the inner electrode of the right photodiode was unbiased. Scale bar for the electrode’s field ranges from 0 V (blue) to 0.2 V (red). Scale bar for the neuron stimulation ranges from 0 mV (black) to 70 mV (white).

It is important to note, however, that pixel size is not the sole factor determining the acuity generated by today’s Euclidean electrodes. One limiter which has the potential to reduce visual acuity is electrode crosstalk (when the field from one electrode stimulates the neurons above a neighboring electrode). However, this can be reduced by surrounding each inner electrode with a grounded outer electrode^34, 35^, such as employed here. To check the extent of the fractal electrodes’ crosstalk, the depolarization of a neuron centered above a neighboring electrode was measured (Fig. 6a). With one of the fractal electrodes (left) set at its operating voltage of 0.32 V and no voltage applied to the adjacent electrode (right), the neighboring neuron was depolarized by 5.7 mV. For identical voltages applied to square electrodes, the neighboring neuron was depolarized by 2.1 mV. In summary, for the voltage at which all nine neurons above the fractal electrode were stimulated and only one was stimulated above the square, the depolarization of the neighboring neuron due to cross talk remained less than half of the 15 mV stimulation condition. This crosstalk could potentially be reduced even further by employing different grounding electrode strategies^35^.

Another limiter is that the Euclidean implants induce glia scarring on their smooth electrode surfaces^36^ which prevents neurons from maintaining close proximity to the electric fields. However, adopting a textured surface reduces glia scarring and keeps neurons in closer proximity to the textured regions^20, 37^. Because the fractal exhibits extensive texture generated by its many inner edges as compared to the square’s four outer edges, we predict the fractal will promote stimulation by ensuring that the neurons are located well within the electrode’s electric field.

Experiments performed on rats with retinal implants reveal a degradation factor of 5.8 in the measured visual acuity when compared to the expected acuity calculated from pixel size^38^. However, this experiment did not employ the local grounded electrodes of our design. Taking into account the minimized crosstalk and potentially reduced glial scarring, we expect the degradation factor of the fractal designs to be less than the 5.8 factor of Euclidean designs, with the precise factor to be quantified by future experiments. However, if we consider a pessimistic scenario and apply a Euclidean degradation factor of approximately 5 to the reduced pixel size of our fractal implants, the predicted acuity is 20/400. In order to gain widespread use, implants must restore vision to ambulatory levels (i.e., those associated with the ability to independently navigate rooms and streets) of 20/400 vision^39^. Our fractal implants therefore offer the first viable approach to restoring vision to ambulatory or better levels.

In the current study, we focused on subretinal implants which stimulate the bipolar neurons located at the retina’s back surface. In our discussions, we assumed the voltage, *V*, was generated by a photodiode^6^ but it could equally be generated by an external voltage source^7^. In either case, the fractal generates an enhanced field leading to greater neuronal stimulation to that achieved by the Euclidean designs considered in this paper. We expect fractal electrodes to also outperform Euclidean geometries in epiretinal implants. However, this would require a lower number of fractal iterations for the following reason. For subretinal implants, the 3 iteration fractal electrode generates a uniform electric field that maximizes stimulation of the bipolar neurons aligned perpendicular to the electrodes. In contrast, epiretinal implants stimulate parallel ganglion axons through a large spatial variation in the electric field^40, 41^. This can be generated by employing the 1 iteration fractal rather than the square electrode (Fig. 2).

In addition, fractal electrodes could be employed for deep brain stimulation, which is being used to address conditions ranging from Parkinson’s disease^42^ to depression^43^, and for prosthetic limbs^44^. In terms of the latter application, it is interesting to contrast our use of fractal electrodes to another study based on larger (5 mm compared to our 20 µm) fractal electrodes designed to stimulate peripheral neurons in the human arm^45^. In our study, we exploit fractal geometry to maximize the electrode’s effective capacitive area *A*_*eff*_ for a constant covering area by embedding repeating patterns within the confined region of a photodiode pixel. In contrast, the limb study employs the repeating patterns to build outward at the expense of losing pixel resolution. The authors show that this large fractal boundary leads to a considerable variation in local charge density, which generates the large spatial variation in the electric field necessary for stimulating peripheral neurons. The two studies demonstrate how fractal geometry can be exploited to achieve very different goals and, taken together, highlight the great promise for future integration of fractal electronics with the human body.

## Methods

**Figure 7:**
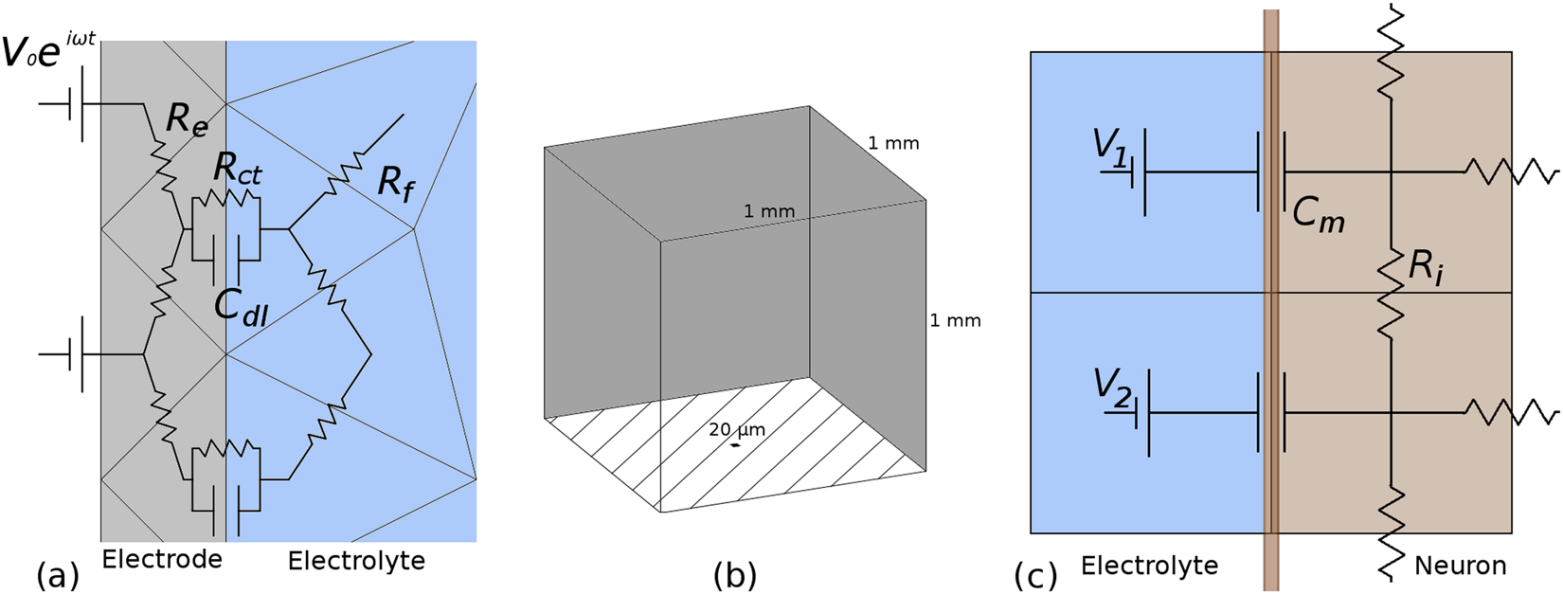
(**a**) Equivalent circuit model with tetrahedral nodes for an electrode surrounded by a conducting electrolyte. (**b**) Boundary conditions for the bounding domain were set to *V* = 0 V on the 5 surfaces far from the center electrode (dark grey) and insulating on the bottom surface (striped). The 20 µm electrode (black) sits on the bottom surface. (**c**) Equivalent circuit model with cubic nodes for a neuron in an electrolyte undergoing high frequency stimulation. The external potential obtained from the electrode-electrolyte simulations was mapped onto the corresponding extracellular neural node, for example, *V*_*1*_ and *V*_*2*_.

An equivalent circuit model was used to solve for the extracellular potential surrounding a TiN electrode (Fig. 7a). Each three-dimensional geometry was first meshed into a set of tetrahedral nodes using COMSOL. The meshes were then exported and the node-to-node impedances defined using custom C code. The fluid-fluid nodes are resistive while the fluid-electrode nodes consist of a capacitive and resistive branch in parallel^46^. The tissue resistivity was taken to be 3,500 Ωcm, the resistivity measured at the photoreceptor layer in macaques monkeys^47^. The specific capacitance of a TiN electrode is 2.5 mF/cm^2, 48^ and the surface resistivity is 3 × 10^5^ Ωcm^2, 49^. The electrode surfaces were assumed to be at an equipotential. A bounding domain of 1 mm^3^ was used for each electrode geometry. The boundary conditions were set to a potential of 0 V on the 5 faces of the cube far away from the electrode and an insulating boundary for the face which the electrode lies on (Fig. 7b).

With the equivalent circuit model established, modified nodal analysis (MNA)^50^ was used to create a system of equations based on solving current conservation equations at each node along with the appropriate boundary conditions. The number of equations was given by the total number of nodes, *n*, plus the number of applied potential source nodes, *m*. The solution output consisted of *n* complex valued potentials and *m* complex valued currents (the complex valued potentials and currents arise from capacitive and resistive components). By applying a sinusoidal potential to the inner electrode, the voltage time derivative for current crossing the electrode-fluid interface, *C*_*dl*_d(*V*_*e*_ − *V*_*f*_)/dt, dropped off, where *V*_*e*_ and *V*_*f*_ are complex valued node voltages of an electrode and fluid element, respectively, and *C*_*dl*_ is the interfacial capacitance. Additionally, since each node oscillated at a frequency, *f*, the e^i2π*ft*^ term could be factored out from the system of equations. The system of equations then formed a sparse matrix which was solved using the linear equations software library, Distributed SuperLU^51, 52^. Up to ~3 million node potentials were solved for. The resulting voltages and currents at differing locations all oscillated with frequency, *f*, but were not necessarily in phase due to varying capacitive and resistive components. Throughout the main text and in Fig. 2, the peak magnitude within each cycle of the extracellular voltage is presented rather than the voltage at a specific time. We note that the extracellular potential at the neuron’s soma is within 1° of phase of the potential at the inner electrode. Although we only tested sinusoidal applied potentials, because any periodic waveform can be written as a sum of sines and cosines, this method could be used, in principle, to apply square waves, triangular waves, or any periodic waveform to the electrode. In addition to determining the potential and currents, the charge density delivered per phase at each node, *Q*_*ph*_, on the electrode surface was also calculated by

1 $$\begin{array}{c}{Q}_{ph}={\int }_{0}^{\frac{1}{2f}}dt{C}_{dl}|\frac{d({V}_{e}\,-\,{V}_{f})}{dt}\,|=2{C}_{dl}|{V}_{e}-{V}_{f}|\end{array}$$

The real and imaginary parts of the extracellular potentials computed from the electrode-electrolyte simulations were then mapped onto the outside membrane of the bipolar neuron (Methods Fig. 1c). We considered extracellular stimulation of passive bipolar neurons (i.e., featuring no voltage-gated ion channels), as has been done previously^53, 54^. Although recent studies indicate voltage-gated transient calcium channels in retinal bipolar neurons are open at the extracellular stimulating frequencies that we operate at, 1000 Hz^55^, calcium current through these open channels is negligible for depolarizations up to ~15 mV^56^. Therefore, the applied voltage, *V*, necessary to depolarize all 9 neurons above an electrode to the Δ*V*_*m*_ = 15 mV condition (Fig. 5) associated with stimulating downstream ganglion neurons^32^ is not affected by our model’s exclusion of voltage-gated channels.

Each neuron was 100 µm in length with a 10 µm diameter soma centered 30 µm above the electrode surface and with 1 µm wide branches^57, 58^. The neuron was first meshed into a set of cubic nodes using MATLAB code. Our custom C code then defined the node-to-node impedance by a capacitive impedance across the neuron membrane and an internal cytoplasmic resistance for the neuron-neuron nodes (Fig. 7c). The passive membrane properties of rod bipolar cells are given by a membrane resistivity *R*_m_ = 2.4 × 10^4^ Ωcm^2^, a membrane capacitance *C*_m_ = 1.1 × 10^−6^ F/cm^2^, and a cytoplasmic resistivity of *R*_*i*_ = 1.3 × 10^2^ Ωcm^59^. The capacitive and resistive components of the membrane impedance are in parallel. We ignored the membrane resistance since the capacitive impedance across the membrane is more than two orders of magnitude lower than the resistive impedance at frequencies of 1 kHz or more. We note that our two-step process of using a tetrahedral grid for the electrode-electrolyte simulations and a cubic grid for the electrolyte-neuron simulations had to be used because a single mesh consisting of electrode, electrolyte and neuron was too complex computationally to be meshed together. The external voltage applied to the neuron was transferred from the electrolyte potential solution to the external neural potential by using the barycentric coordinate formula to determine which tetrahedral node the cubic node fell into. From here, another MNA matrix was set up and solved using Distributed SuperLU. The solution contains the complex valued potential at each node in the model neuron.

## Electronic supplementary material

Supplementary
