# Supplementary material for: Fractal Electrodes as a Generic Interface for Stimulating Neurons
Source: Sci Rep. 2017 Jul 27;7:6717. doi: 10.1038/s41598-017-06762-3 (PMC5532230; doi:10.1038/s41598-017-06762-3)
Supplement: Supplementary file 1 — Supplementary [file 41598_2017_6762_MOESM1_ESM.pdf]

Fractal Electrodes as a Generic Interface for Stimulating Neurons

Supplementary Information

W.J. Watterson,* R.D. Montgomery and R.P. Taylor

Physics Department, University of Oregon, Eugene, OR 97403, USA

*corresponding author (email: wwatters@uoregon.edu)

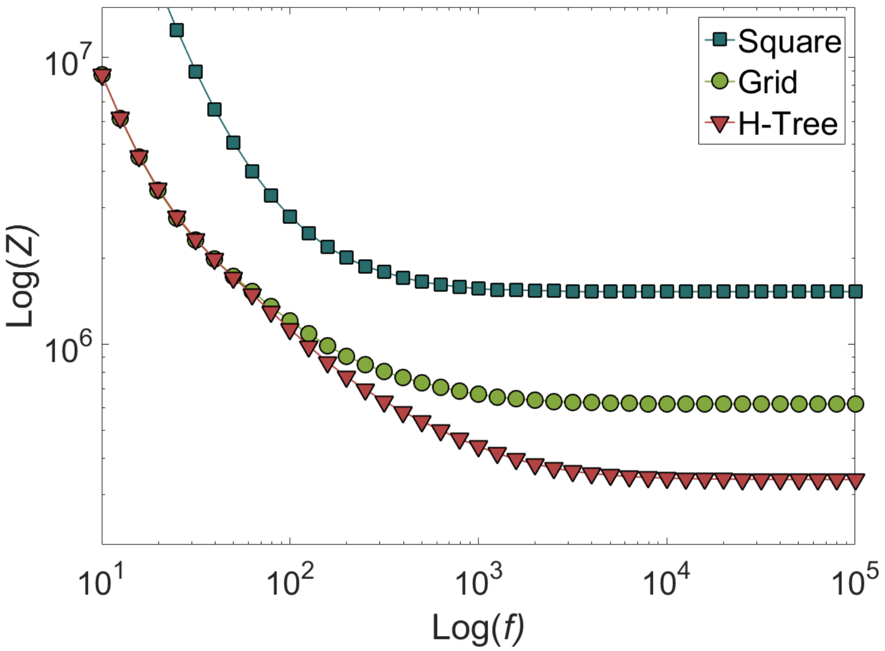

Supplementary Figure 1. Impedance *Z* plotted against oscillation frequency *f* for square, grid, and fractal H-tree geometries. At large *f*, *Z* is inversely proportional to the bounding area of the electrode (total area enclosed by the white dashed lines in Fig. 1).

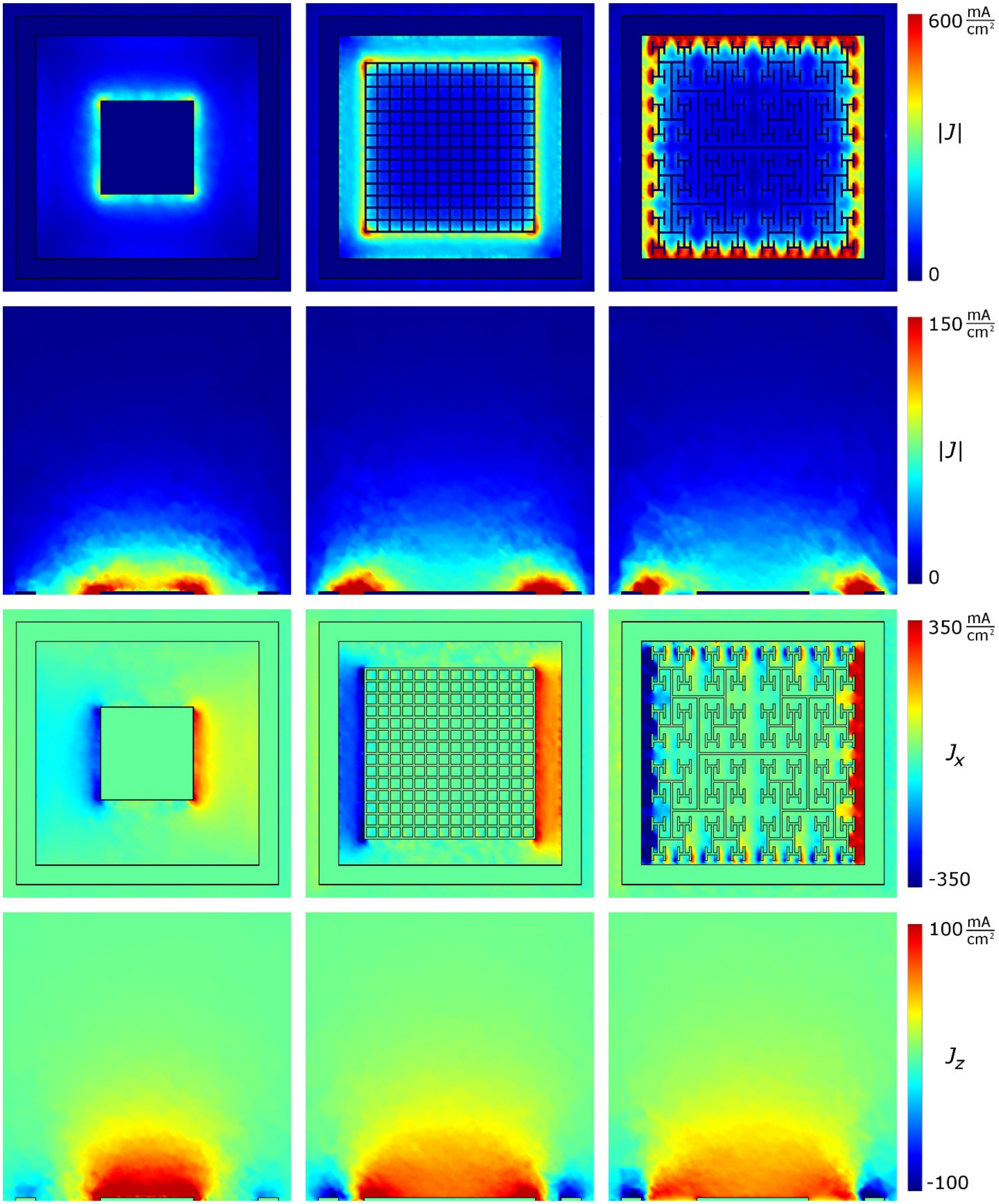

Supplementary Figure 2. Current density J surrounding the square, grid, and H-tree electrodes occurring at the electrode's maximum voltage within each oscillation for an applied voltage with V_0 = 0.2 V and f = 1 kHz. First and second rows show the current density magnitude for a horizontal slice at the inner electrode's surface and a vertical slice half way through the electrode, respectively. The third row shows the horizontal component of the current density J_x at the inner electrode's surface (where x is the left-right direction). The fourth row shows the vertical component of current density J_z at the slice half way through the electrode.

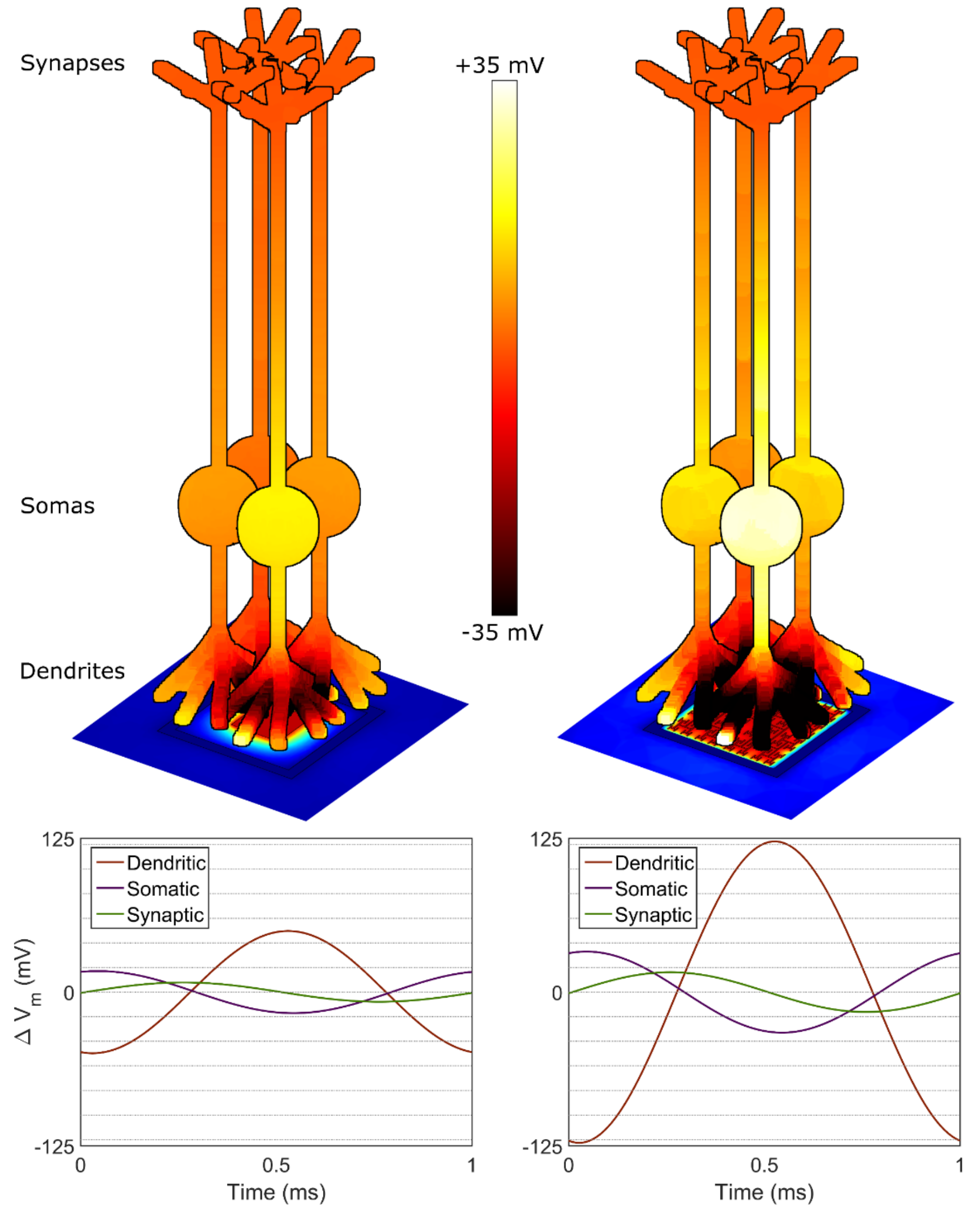

Supplementary Figure 3. Top: Change in membrane potential, ΔV_m, for a patch of 4 bipolar neurons at the time of maximum somatic depolarization for the square (left) and H-tree (right) electrodes. In each case, this maximum somatic ΔV_m occurs during the positive phase of applied electrode voltage. For both the square and fractal electrodes at this time in the cycle, the neuron's soma and synapses are depolarized while the dendrites are hyperpolarized (i.e., $\Delta V_m < 0$). Bottom: ΔV_m is compared at three locations for the central neuron (dendritic, somatic, and synaptic) as a function of time during the electrode's oscillation. For the fractal, the peak synaptic depolarization leads the peak somatic depolarization by 77° degrees of phase while for the square it leads by 78°.

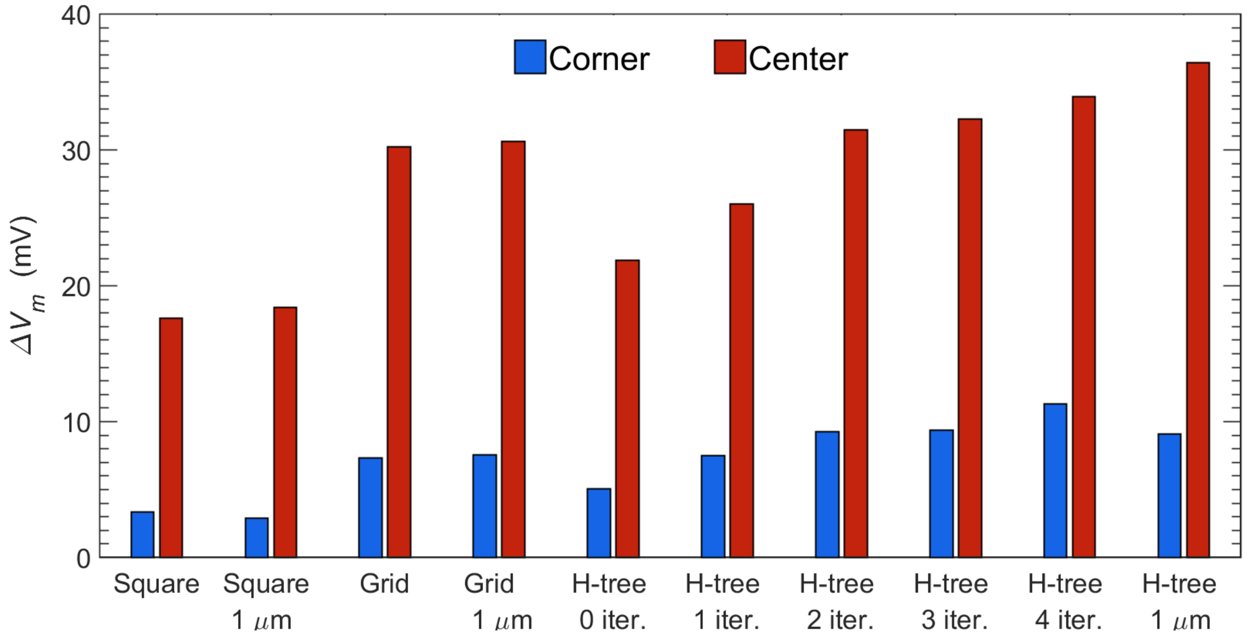

Supplementary Figure 4. ΔV_m for neurons located directly above the electrode for each electrode geometry with V_0 = 0.2 V and f = 1 kHz. Neurons positioned above the electrodes' center are represented in red, while those positioned above the electrode's corners are shown in blue. The associated extracellular potentials are shown in main text Fig. 2. There is a general trend towards greater depolarization as more iterations are added into the H trees. There is also a trend of increased depolarization for the center neuron above 1 μm electrodes (compared to 250nm for the other bars).
